# Loss of *Dlg-1* in the Mouse Lens Impairs Fibroblast Growth Factor Receptor Signaling

**DOI:** 10.1371/journal.pone.0097470

**Published:** 2014-05-13

**Authors:** SungKyoung Lee, Anne E. Griep

**Affiliations:** Department of Cell and Regenerative Biology, University of Wisconsin-Madison, Madison, Wisconsin, United States of America; University of Delaware, United States of America

## Abstract

Coordination of cell proliferation, differentiation and survival is essential for normal development and maintenance of tissues in the adult organism. Growth factor receptor tyrosine kinase signaling pathways and planar cell polarity pathways are two regulators of many developmental processes. We have previously shown through analysis of mice conditionally null in the lens for the planar cell polarity gene (PCP), *Dlg-1,* that *Dlg-1* is required for fiber differentiation. Herein, we asked if *Dlg-1* is a regulator of the Fibroblast growth factor receptor (Fgfr) signaling pathway, which is known to be required for fiber cell differentiation. Western blot analysis of whole fiber cell extracts from control and *Dlg-1* deficient lenses showed that levels of the Fgfr signaling intermediates pErk, pAkt, and pFrs2α, the Fgfr target, Erm, and the fiber cell specific protein, Mip26, were reduced in the *Dlg-1* deficient fiber cells. The levels of Fgfr2 were decreased in *Dlg-1* deficient lenses compared to controls. Conversely, levels of Fgfr1 in *Dlg-1* deficient lenses were increased compared to controls. The changes in Fgfr levels were found to be specifically in the triton insoluble, cytoskeletal associated fraction of *Dlg-1* deficient lenses. Immunofluorescent staining of lenses from E13.5 embryos showed that expression levels of pErk were reduced in the transition zone, a region of the lens that exhibits PCP, in the *Dlg-1* deficient lenses as compared to controls. In control lenses, immunofluorescent staining for Fgfr2 was observed in the epithelium, transition zone and fibers. By E13.5, the intensity of staining for Fgfr2 was reduced in these regions of the *Dlg-1* deficient lenses. Thus, loss of *Dlg-1* in the lens impairs Fgfr signaling and leads to altered levels of Fgfrs, suggesting that *Dlg-1* is a modulator of Fgfr signaling pathway at the level of the receptors and that *Dlg-1* regulates fiber cell differentiation through its role in PCP.

## Introduction

Coordination of cell proliferation, differentiation and survival is essential for normal development and maintenance of tissues in the adult organism. This coordination involves growth factor receptor tyrosine kinase signaling pathways that they activate, as these pathways are critical regulators of cell fate [1], [2]. Cell structure and the coordinated movement of cells during differentiation are also determinants of normal development. One property governing the coordinated movement of cells is referred to as Planar Cell Polarity (PCP), the polarized orientation of cells within a plane of a tissue perpendicular to the apical-basal axis [3], [4], [5]. In this study, we address the possibility that *Dlg-1*, a newly discovered PCP gene, is a regulator of the Fibroblast growth factor receptor (Fgfr) signaling pathway in the terminal differentiation of the epithelial cells in the mouse ocular lens.

The mouse ocular lens has been widely used as a system for studying mechanisms of mammalian development in part because of its spatially separated regions of cycling and differentiating cells. Terminal differentiation of lens epithelial cells into fiber cells involves cell cycle withdrawal, cytoskeletal reorganization, directed cell migration and organelle loss, all of which must be tightly coordinated [6]. In the mouse, lens fiber cell differentiation begins after the formation of the hollow lens vesicle at day E10.5. Cells in the posterior of the lens vesicle, closest to the developing retina, withdraw from the cell cycle, begin to express fiber cell specific proteins and elongate toward the anterior surface to form the primary fiber cells. Once the vesicle is occluded, around day E12.5, secondary fiber cells begin to differentiate from the periphery of the lens as epithelial cells withdraw from the cell cycle, elongate and migrate along the capsule and anterior epithelium until they meet up with their counter part from the opposite side of the lens to form the lens sutures [6].

The vitreous humor of the eye, which fills the space between the posterior of the lens and the retina, contains various growth factors including epidermal growth factor (EGF), fibroblast growth factor (FGF), insulin-like growth factor (IGF), platelet-derived growth factor (PDGF), hepatocyte growth factor (HGF), and vascular endothelial growth factor (VEGF) [1], [7]. Among these various growth factors, FGF has been demonstrated to be the only growth factor capable of initiating lens fiber differentiation [7], [8], [9]. Studies using a rat lens explant system have demonstrated that a low dose of FGF can induce proliferation, a medium dose can lead to cell migration, and a high dose can elicit differentiation, as measured by cell elongation and expression of lens fiber cell specific proteins. FGFs affect cell behavior through the activation membrane bound tyrosine kinase receptors and the signaling pathways regulated by those receptors. Four Fgfrs are expressed in the embryonic mouse lens, *Fgfr1*, *Fgfr2, Fgfr3,* and *Fgfr4* [2], [10], [11], [12]. *In vivo* experiments using transgenic mice have shown that overexpression of numerous FGFs can elicit premature fiber cell differentiation whereas overexpressing a dominant-negative Fgfr inhibit lens fiber differentiation [13], [14], [15]. And, importantly, gene knockout experiments have shown that the simultaneous deletion of *Fgfrs* 1, 2 and 3 once the lens vesicle has formed results in complete inhibition of fiber cell differentiation [16], demonstrating that Fgfr signaling is required for fiber cell differentiation.

How the Fgfr signaling pathway and the changes in cellular shape and organization that occur during fiber cell differentiation are coordinated is not well understood. However, recently, it has been shown that the newly forming fiber cells in the outermost regions of the fiber cell compartment exhibit planar cell polarity and lens fiber cells in mice carrying mutations in the core PCP gene, *Vangl2*, exhibit orientation, migration and suture defects [17], [18]. Furthermore, transgenic expression of an inhibitor of Wnt signaling, secreted frizzled related protein 2 (Sfrp2) inhibited fiber cell differentiation [17]. Finally, it has been shown that FGF-induced fiber cell differentiation in rat explant cultures is dependent on Wnt signaling [19]. All these studies point toward a requirement for Wnt/PCP signaling in the transmission of Fgfr signaling to induce fiber cell differentiation.

Recently, we discovered that *Discs large-1* (*Dlg-1)*, the mouse homolog of the *Drosophila* gene *discs-large* (*dlg),* is expressed in the lens [20], [21] and, using lens specific deletion of *Dlg-1*, we demonstrated that *Dlg-1* is required for cell adhesion, apical-basal polarity and fiber cell differentiation in the lens [22], consistent with the role documented for *dlg* in *Drosophila* [23]. However, in addition, the phenotype of the *Dlg-1* deficient lens resembled that described for the *Vangl2^Lp/Lp^* mutant lens. In histological section, the shape of the *Dlg-1* mutant lens was flattened, fiber cell curvature remained concave rather than becoming convex and defects were observed in suture formation. The similarity in the phenotype of the *Vangl2^Lp/Lp^* and *Dlg-1* mutant lenses suggested that *Dlg-1* might also play a role in PCP. Consistent with the prediction that *Dlg-1* might play a role in PCP, we found that mice carrying germline mutation in *Dlg-1* show characteristic phenotypes of mice deficient for core PCP genes [24], thereby identifying *Dlg-1* as a new PCP gene.

In the course of our analysis of the phenotype of the *Dlg-1* deficient lens, we observed that levels of activated Erk, a signaling intermediate of numerous growth factor signaling pathways, were reduced in the *Dlg-1* deficient fiber cells [22]. Erk activation downstream of FGF stimulation is necessary for lens proliferation [25], and differentiation [26]. This led us to hypothesize that loss of *Dlg-1* function in the lens impairs the Fgfr signaling pathway resulting in the observed fiber cell differentiation defects. In this study, we tested this hypothesis. Specifically, we assessed the impact of loss of *Dlg-1* on components of the Fgfr signaling cascade, an Fgfr signaling target, and on Fgfrs 1, 2, and 3, themselves. We found that activation of signaling intermediates in the Fgfr pathway, along with levels of an Fgfr target, were reduced in *Dlg-1* deficient lenses. *Dlg-1* deficiency also led to changes in the relative levels of Fgfrs 1, 2 and 3. Finally, reduced levels of Fgfr2 and pErk were observed in the transition zone of embryonic lenses, a region of the lens that exhibits PCP. Taken together, these data show that *Dlg-1* is required for the proper levels of Fgfrs and Fgfr signaling, and suggest that *Dlg-1* acts as modulator of fiber differentiation in the lens through its role in PCP.

## Materials and Methods

### Ethics Statement

All procedures using mice conformed to the Guide for the Care and Use of Laboratory Animals of the National Institutes of Health and the ARVO Statement for the Use of Animals in Ophthalmic and Vision Research. The protocol covering these studies (protocol #M00712) was approved by the Institutional Animal Care and Use Committee of the University of Wisconsin School of Medicine and Public Health (Animal Welfare Assurance #A3368-01).

### Animals and Tissue Preparation

The generation of mice carrying the *Dlg-1* conditional allele (*Dlg-1^f/f^* mice) [22] and the Cre driver transgenic mice, *MLR10CRE* and *MLR39CRE* [27] have been described previously. *Dlg-1^f/f^* mice were crossed to either *MLR10CRE* or *MLR39CRE* mice to generate *Dlg-1^f/f^;MLR10CRE* mice (here after referred to as *Dlg10CRE* mice) or *Dlg-1^f/f^;MLR39CRE* mice (here after referred to as *Dlg39CRE* mice). Genotyping was carried out as described previously [22]. Heads from embryonic day 12.5 (E12.5), E13.5, E15.5 and E17.5 control (*Dlg-1^f/f^*), *Dlg10CRE*, and *Dlg39CRE* embryos were fixed in 4% paraformaldehyde overnight at 4°C, washed in 1X phosphate buffered saline (PBS), dehydrated in increasing concentrations of ethanol, and embedded in paraffin oriented for longitudinal sectioning. Sections (5 µm) were cut and used for immunofluorescence. Embryos were staged by designating midday on the day of the vaginal plug as day E0.5. Postnatal mice were staged by designating the day of birth as neonate and subsequent days as postnatal day 1 (P1), P2, etc. Eyes from P30 control mice were fixed in 4% paraformaldehyde for 2 hours at 4°C, incubated in 10% and 20% sucrose for 1.5 hour each at 4°C and then in 30% sucrose overnight at 4°C and embedded in TissueTek oriented for transverse sectioning. Cryogenic sections (10 µm) were cut and used for immunofluorescence.

### Western Blot

Fiber cells were dissected from postnatal day 2 (P2) control, *Dlg10CRE, Dlg39CRE* mice and protein lysates were prepared by extraction in RIPA buffer with protease inhibitors. A total of 50–100 µg of each protein lysate was electrophoresed through 7.5% acrylamide gels, and the proteins were transferred to PVDF membranes. Membranes were blocked for one hour at room temperature (RT) in 5% nonfat dry milk dissolved in 1X phosphate buffered saline-tween 20 (PBST) or 5% bovine serum albumin (BSA) in Tris buffered saline-tween 20 (TBST). Blots were incubated with rabbit anti-human MIP26 (Alpha Diagnostics cat #AQP01-A) at 1∶100 dilution, rabbit anti-human total ERK (Promega cat #V1141) at 1∶100 dilution, rabbit anti-human pERK (Cell Signaling cat #4370) at 1∶100 dilution, rabbit anti-human pAKT (Abcam cat #ab66138) at 1∶1000 dilution, rabbit anti-human pFRS2α (Cell Signaling cat #3861) at 1∶100 dilution, and rabbit anti-human ERM (Santa Cruz cat #SC-22807) at 1∶1000 dilution were incubated overnight at 4°C. Membranes were washed in 1X PBST or 1X TBST and were then incubated for one hour at RT with goat anti-mouse horseradish peroxidase (HRP, Pierce) or donkey anti-rabbit HRP (GE Healthcare Life Sciences) diluted in blocking solution. To prepare soluble and cytoskeletal associated protein fractions, lenses from P2 control and *Dlg10CRE* lenses were lysed in Triton X-100 buffer (1% Triton X-100, 10 mM imidazole, 100 mM NaCl, 1 mM MgCl_2_, 5 mM EDTA) and centrifuged at 13,200 rpm for 15 minutes at 4°C to generate the detergent soluble supernatant (cytoplasmic) and detergent insoluble pellet (cytoskeletal associated) fractions. The pellets were resuspended in Urea buffer (7 M urea, 0.1 M Tris, pH 7.9, 5 mM EDTA) and centrifuged. A total of 50 µg of each soluble and 100 µg of each insoluble protein lysate was electrophoresed, transferred and immunoblotted for Fgfrs using rabbit anti-human FGF receptor 1, 2, or 3 antibodies (Santa Cruz Biotechnology cat #s SC-7945, SC-122, and SC-123, respectively) at 1∶100 dilution as described above. All blots were reprobed with mouse anti-rabbit GAPDH antibody (Millipore cat #MAB374) as a loading control. Bands were visualized using Enhanced Chemiluminescence Plus kit (ECL plus, GE Healthcare Lifesciences) and protein levels were quantified by phosphorimager analysis on a Storm Scanner. At least three pools were generated and each pool was analyzed in triplicate over 1–3 blots. Relative protein levels were calculated by setting the protein/Gapdh ratio for the controls at 1.0. All data for an individual pool were combined to give a single value for the pool. The data reported are the mean ± standard deviation across 3–4 pools. For statistical analysis, the two-sided One Sample t-test was carried out using MSTAT software (www.mcardle.wisc.edu/mstat). Because of the large number of experimental comparisons made in these studies, we report the false discovery rates [28] rather than the unadjusted per comparison P-values from the statistical tests. A false discovery rate (FDR) ≤0.05 was considered to be statistically significant.

### Immunofluorescence

Heads from day E12.5, E13.5, E15.5, and E17.5 control, *Dlg10CRE* or *Dlg39CRE* embryos were fixed, embedded in paraffin, and sectioned as described above. For immunofluorescence detection, sections were deparaffinized in xylenes and rehydrated using graded ethanols. For Fgfr2 immunofluorescent staining, sections were trypsinized for 40 minutes at RT in a humidified chamber, washed in 1X PBS, and then incubated for 1 hour with a 0.5% Triton X-100 and 0.3 M glycine in 1X PBS in a humidified chamber. After antigen retrieval, sections were blocked in 0.5% nonfat dry milk, 10% horse serum, and 0.2% Triton X-100 diluted in 1X PBS for 3 hours at RT. For pErk and Dlg-1 immunofluorescent staining, antigen retrieval was accomplished by boiling the sections in 0.1 M sodium citrate, pH 6.0, for 30 minutes in a rice cooker and then washed in 1X PBS. Sections were blocked with 5% horse serum in 1X PBS for 1 hour. The blocking solution was removed and sections were incubated with rabbit anti-human FGFR2 (Abcam cat#ab10648 which was raised against amino acids 362–374 in the extracellular domain of human FGFR2) diluted in blocking solution at 1∶200 dilution, rabbit anti-human pERK (Cell Signaling cat#4370) diluted at 1∶50, or mouse anti-rat SAP97 (Novus cat#NBP1-48054) at 1∶500 overnight at 4°C. Sections were washed in 1X PBS and were incubated with the AlexaFluor 568 conjugated goat anti-rabbit (Molecular Probes) or FITC-conjugated horse anti-mouse (Vector Labs) for 1 hour at RT. Sections were counterstained with To-Pro3 (Invitrogen cat#T3605) to visualize nuclei. Stained sections viewed by confocal microscopy. Fluorescence intensities in the regions of the lenses were quantified by drawing a box around the area (epithelium, transition zone, anterior region of fiber cells) and measuring the intensity using ImageJ. Fluorescence intensities in the corresponding retinae were also measured and used as internal controls. At least 3 sections over two slides for at least 3 eyes per time point were analyzed. The relative Fgfr2 or pErk levels were calculated by setting the lens/retina value for the controls at 1. The data for each eye was combined to give a single value for the eye. The data reported are the mean ± standard deviation across the samples for each time point. For statistical analysis, the two-sided One Sample t-test was conducted using MSTAT software. A FDR≤0.05 was considered to be statistically significant. To verify the specificity of the FGFR2 antibody, lens sections from control and *Fgfr2^f/f^;LeCre* E17.5 embryos were subjected to immunofluorescence analysis and the signal intensities quantified (samples provided by Dr. Michael L. Robinson, Miami University, Oxford, OH). Stained *Fgfr2* conditional null lens sections showed a greater than 90% reduction in signal intensity compared to control lens sections (Fig. S1). For immunofluorescent staining of P30 cryogenic sections, sections were trypsinized for 45 minutes at room temperature, washed twice in PBS, blocked with 10% horse serum in 0.5% Triton X-100 for 1 hour at RT, incubated with mouse anti-rat SAP97 diluted at 1∶500 in 10% horse serum and anti-human FGFR2 diluted at 1∶200 in 10% horse serum overnight at 4°C. Sections were washed in 1X PBS and were incubated with the AlexaFluor 568 conjugated goat anti-rabbit (Molecular Probes) and FITC-conjugated horse anti-mouse (Vector Labs) for 1 hour at RT.

## Results

### Effect of Loss of *Dlg-1* on Activation of the Fgfr Signaling Pathway

**Figure 1:**
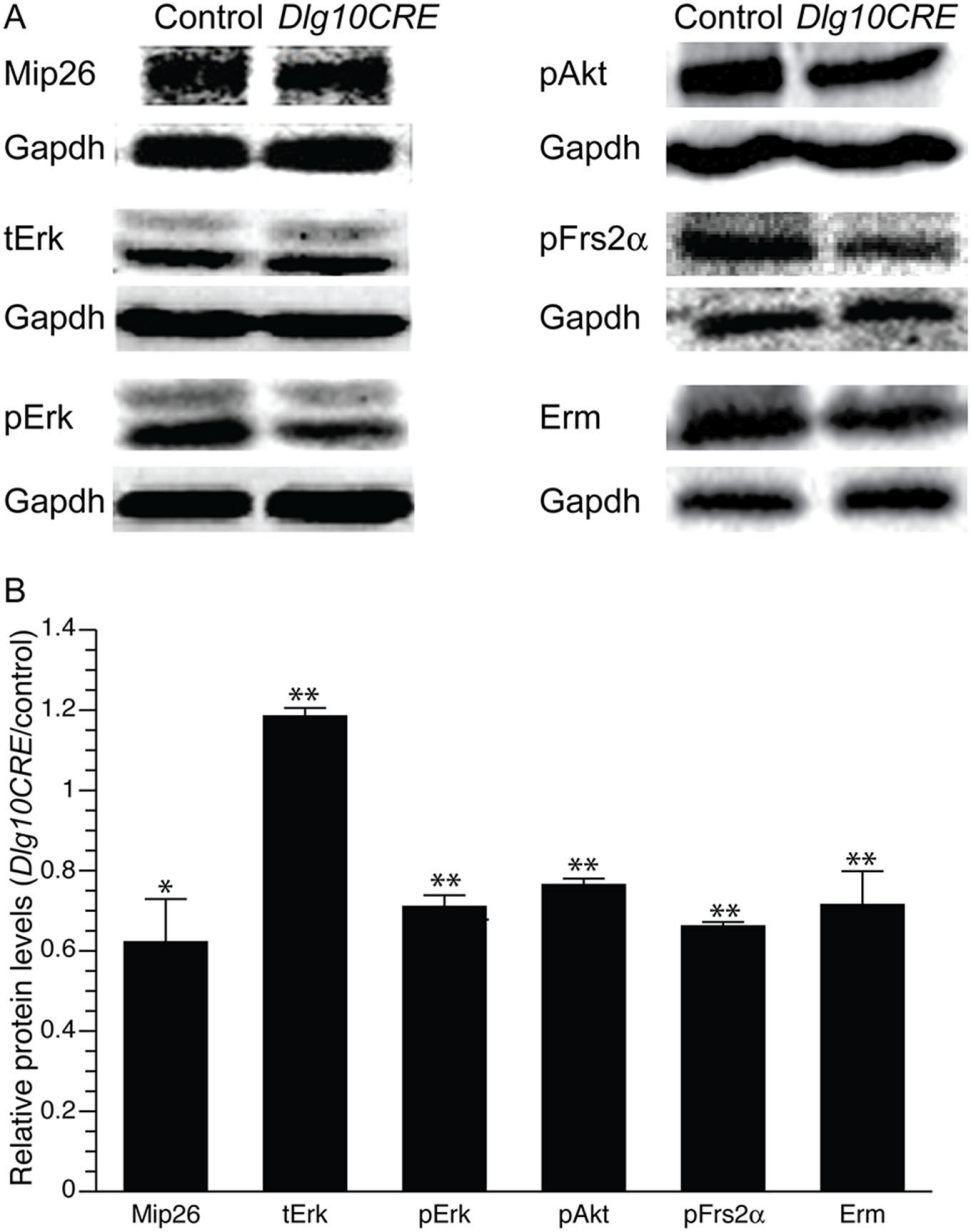
Components of the Fgfr signaling pathway are reduced in *Dlg10CRE* fiber cells. (A) RIPA lysates from P2 control and *Dlg10CRE* fiber cells were immunoblotted for the indicated proteins and the blots reprobed for Gapdh as a loading control. Representative blots are shown. (B) Quantification of protein levels. Shown are the levels of the indicated proteins in extracts from *Dlg10CRE* fiber cells relative to levels in the controls (control levels set a 1.0). Signal intensities were quantified by phosphorimager analysis, as described in Materials and Methods, and the data subjected to statistical analysis using the two-sided One Sample t-test. At least 3 protein pools were blotted in triplicate over 1–3 blots. The relative levels of Fgfr signaling intermediates and target as well as the fiber cell specific protein, Mip26, were all reduced in *Dlg10CRE* lens fibers as compared to control. Error bars = standard deviation. * = FDR<0.05, ** = FDR<0.01.

We previously have shown that ablation of *Dlg-1* in the lens leads to defects in fiber cell differentiation including reduced levels of the fiber cell specific protein, Mip26. Additionally, levels of activated Erk (pErk), a signaling intermediate in the Ras/Raf/Mek/Erk pathway that is activated by various growth factor signaling pathways including the Fgfr pathway [1], [2] appeared to be reduced in *Dlg-1* deficient fiber cells [22]. Because Fgfr signaling has been shown to be essential for fiber cell differentiation [2], [14], we hypothesized that Fgfr signaling is compromised in *Dlg-1* deficient lenses. To test this hypothesis, *Dlg-1^f/f^* mice, which carry a conditional null allele of *Dlg-1*, were crossed to *MLR10CRE* mice [22] to generate *Dlg-1^f/f^* mice (controls) and *Dlg10CRE* mice in which *Dlg-1* is ablated throughout the lens beginning at day E10.5 in development. Whole cell lysates were prepared from lens fiber cells of postnatal day 2 (P2) mice control and *Dlg10CRE* mice and the lysates subjected to western blot analysis using anti-MIP26 and anti-pERK antibodies. The blots were subsequently reprobed using anti-GAPDH antibody as a loading control and the levels quantified by phosphorimager analysis. Levels of Mip26 were reduced by 40% in *Dlg10CRE* lenses as compared to controls and levels of pErk were reduced by 30%, despite an increase in levels of total Erk (tErk; Fig. 1). Fgfr signaling is also known to activate another signaling intermediate, pAkt, via the PI3K pathway [1]. Levels of pAkt, were reduced by 25% in *Dlg10CRE* fiber cell extracts as compared to controls (Fig. 1).

Fgfr signaling stimulates the activation of signaling cascades starting with the phosphorylation of the docking protein, Frs2α [29], [30], which binds directly to the receptor. Although Frs2α can be activated by receptors in addition to Fgfrs, these other receptors are not expressed in the lens [31]. Thus, activation of Frs2α in the lens is a read out specifically for Fgfr signaling. To determine if loss of *Dlg-1* affected the levels of Frs2α activation, western blot analysis were carried out on P2 fiber cell extracts from control and *Dlg10CRE* lenses using anti-pFrs2α antibodies. Levels of pFrs2α were decreased by 30% in extracts from *Dlg10CRE* lenses as compared to controls. Finally, western blot analysis showed that protein levels of Erm, an Fgfr target, were decreased by 30% in extracts from *Dlg10CRE* fiber cells as compared to fiber cells from controls. Thus, levels of activated Fgfr signaling intermediates, including the proximal Frs2α, an Fgfr target, Erm, and a fiber cell marker, Mip26, were all significantly reduced in fiber cell extracts from *Dlg10CRE* mice, indicating that *Dlg-1* is a modulator of Fgfr signaling and fiber cell differentiation in the lens.

**Figure 2:**
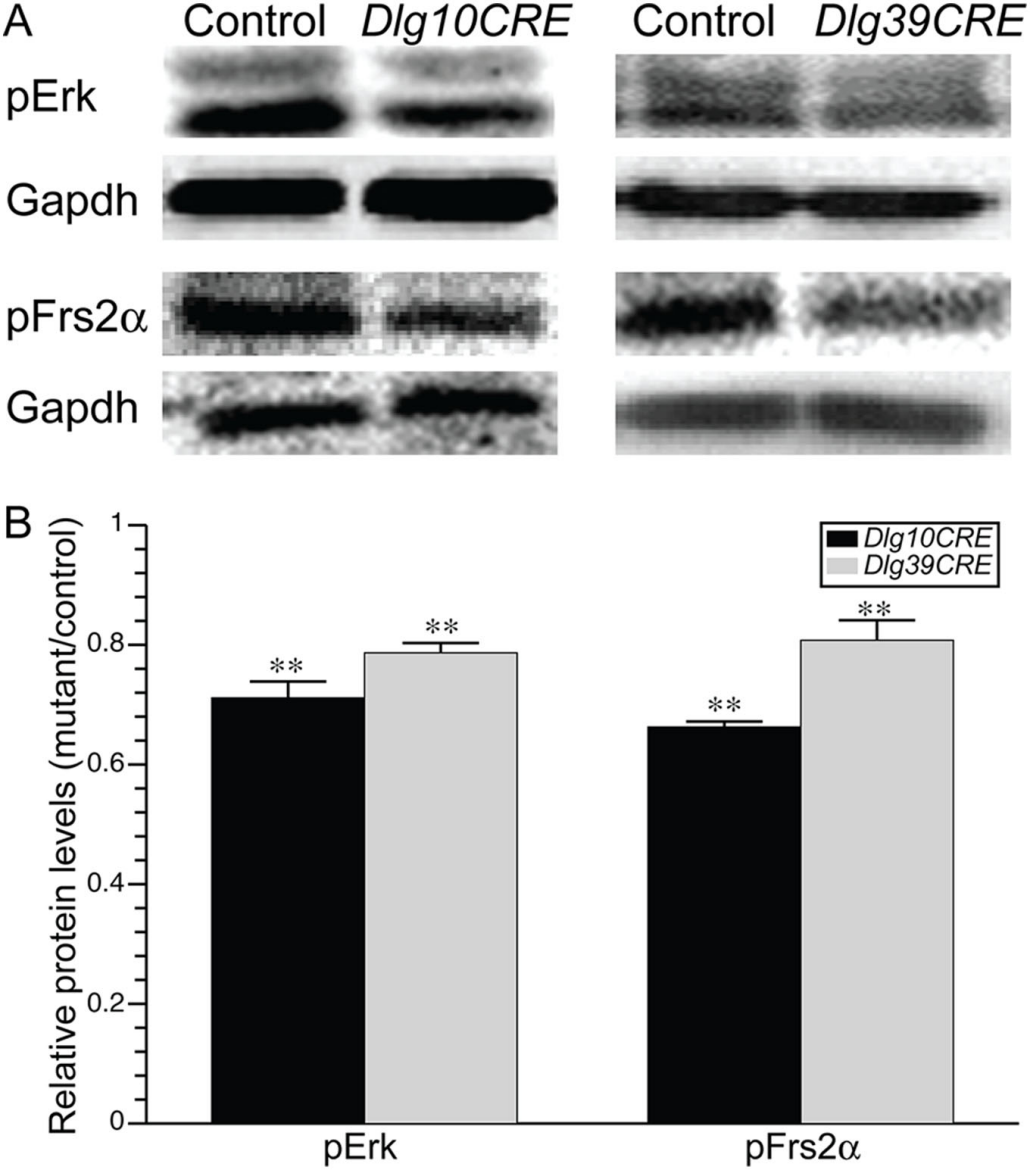
Levels of Fgfr signaling intermediates are reduced in both *Dlg10CRE* and *Dlg39CRE* fiber cells. (A) RIPA lysates from P2 control, *Dlg10CRE*, and *Dlg39CRE* fiber cells were immunoblotted for the pErk and pFrs2α and the blots reprobed for Gapdh as a loading control. Representative blots are shown. (B) Quantification of protein levels. Shown are the levels of the indicated proteins in extracts from *Dlg10CRE* fiber cells relative to levels in the controls (control levels set at 1.0). Signal intensities were quantified by phosphorimager analysis, as described in Materials and Methods, and the data subjected to statistical analysis using the two-sided One Sample t-test. At least 3 protein pools were blotted in triplicate over 1–3 blots. The relative levels of Fgfr signaling intermediates were reduced in the fiber cells of *Dlg39CRE* mice as well as *Dlg10CRE* mice compared to controls, indicating that the effect was fiber cell autonomous. Error bars = standard deviations. ** = FDR<0.01.

In *Dlg10CRE* mice, *Dlg-1* is ablated in both epithelial and fiber cells. Previously, by comparing the phenotype of the fiber cells in these mice to mice in which *Dlg-1* is ablated only in the fiber cells (referred to as *Dlg39CRE* mice), we showed that the epithelial and fiber cell defects were independent of each other [22]. To verify that the effects of *Dlg-1* deficiency on Fgfr signaling in the fiber cells is a fiber cell autonomous effect, we quantified the levels of pErk and pFrs2α in the fibers of *Dlg39CRE* mice as compared to *Dlg10CRE* mice and controls by western blot analyses. As shown in Fig. 2, levels of pErk and pFrs2α were significantly reduced in the fiber cells from *Dlg39CRE* mice as compared to controls (20% reduction in each protein), although to a lesser extent than in the fiber cells from *Dlg10CRE* mice (30% reduction in pErk and 35% reduction in pFrs2α). That the reduction in pErk and pFrs2α in *Dlg39CRE* fibers is less than in *Dlg10CRE* fibers likely is due to a delay in the timing of Cre activity and/or in the turnover of existing Dlg-1 protein relative to the initiation of fiber cell differentiation [27]. These data indicate that the effect of *Dlg-1* deficiency on Fgfr signaling is fiber cell autonomous.

### Effect of Loss of *Dlg-1* on Levels of Fgfrs

**Figure 3:**
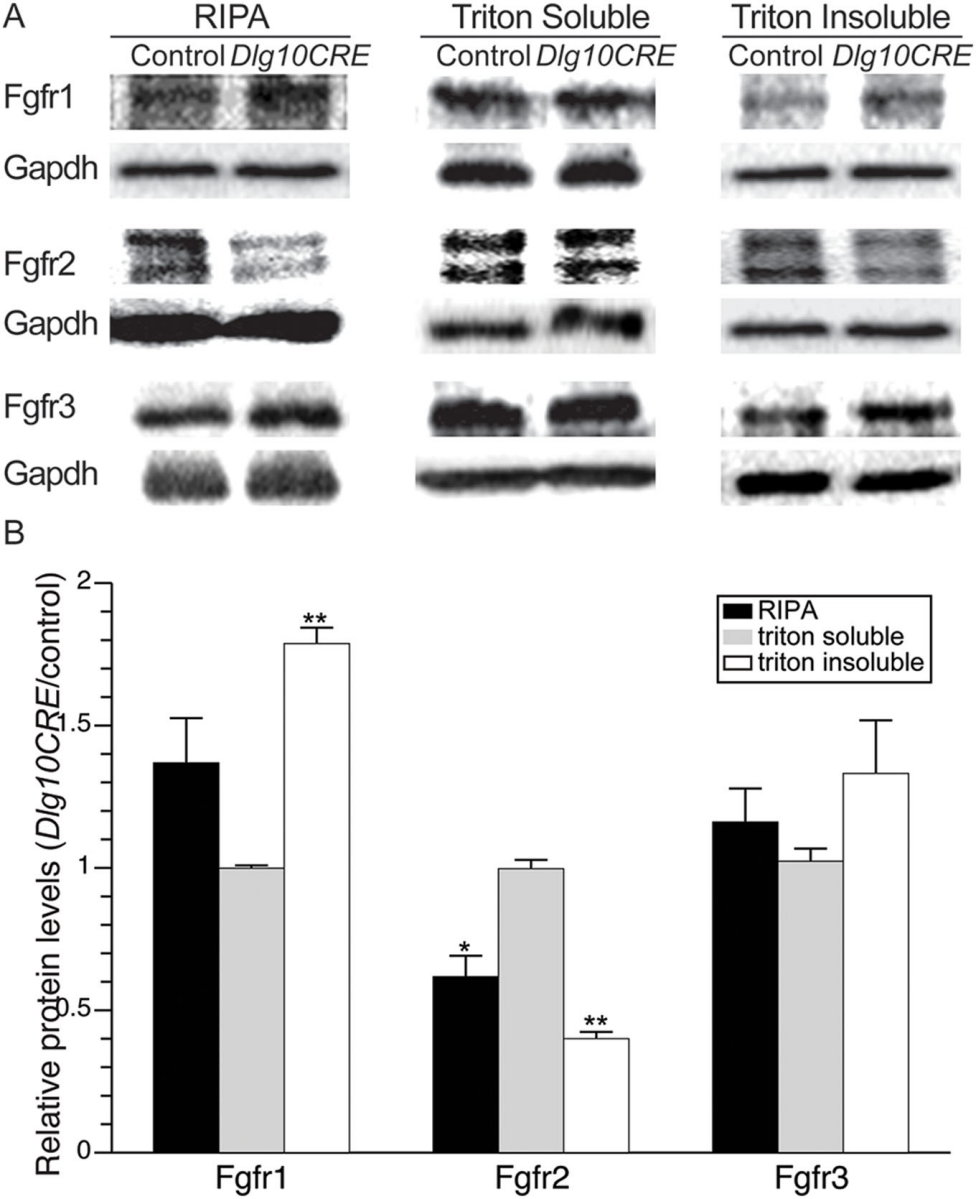
Levels of Fgfrs are altered in the lenses of *Dlg10CRE* mice. (A) RIPA, triton soluble (cytosolic) and triton insoluble (cytoskeletal-associated) extracts from P2 control and *Dlg10CRE* lenses were subjected to western blot analysis for Fgfr1, Fgfr2, and Fgfr3 and the blots reprobed for Gapdh as a loading control. Representative blots are shown. (B) Quantification of protein levels. Shown are the levels of each Fgfr in extracts from *Dlg10CRE* lenses relative to levels in the control (control levels set at 1.0). Signal intensities were quantified by phosphorimager analysis, as described in Materials and Methods, and the data subjected to statistical analysis using the two-sided One Sample t-test. At least 3 protein pools were analyzed in triplicate over 1–3 blots. The relative levels of Fgfr2 were reduced in the whole cell extract and cytoskeletal associated fraction compared to controls whereas the levels of Fgfr1 were increased as compared to controls. Error bars = standard deviations. * = FDR<0.05, ** = FDR<0.01.

The reduced levels of activated Frs2α, which is phosphorylated directly by Fgfr, suggested that the levels of Fgfrs themselves might be altered in the lenses of *Dlg10CRE* mice. To address this possibility, whole cell extracts from P2 fiber cells of *Dlg10CRE* and control mice were subjected to western blot analyses using antibodies specific to Fgfr1, Fgfr2 or Fgfr3. Levels of Fgfr2 were significantly reduced by 40% in *Dlg10CRE* lenses compared to controls; however, levels of Fgfr1 and Fgfr3 trended toward being increased in the *Dlg-1* deficient lenses (Figs. S2 and 3). To understand if the subcellular distribution of Fgfrs were affected, lenses from P2 control and *Dlg10CRE* mice were extracted in Triton X-100 to generate Triton X-100 soluble and insoluble fractions. The triton soluble fraction contains mainly cytosolic proteins while the triton insoluble fraction contains cytoskeletal proteins, membrane proteins, and cytosolic proteins that are tightly associated with the membrane. Immunoblotting the triton soluble and insoluble fractions for Mip26, an intrinsic membrane protein, confirmed that the fractionation was successful as the amount of Mip26 in the soluble fraction was less than 2% of that in the insoluble fraction (data not shown). Interestingly, the levels of Fgfrs were altered in the insoluble fraction from the *Dlg10CRE* lenses, relative to control lenses whereas the levels of soluble fractions of *Dlg10CRE* were similar to the levels in controls. The Fgfr2 levels were reduced by 60% in the cytoskeletal-associated fraction of *Dlg10CRE* lenses. Fgfr1 was significantly increased by 75% in *Dlg10CRE* insoluble fractions and levels of Fgfr3 were increased, although the increase was not statistically significant (Fig. 3). These results demonstrate that *Dlg-1* is required to maintain the normal levels of Fgfrs in the lens and thereby regulate Fgfr signaling.

### Effect of Loss of *Dlg-1* on Perk Levels during Embryogenesis

The western blot analyses demonstrating that Fgfr signaling is impaired in *Dlg-1* deficient lenses were obtained from lens fibers from P2 mice. However, fiber cell differentiation begins during embryogenesis. Primary fiber cells begin to differentiate once the lens vesicle has detached from the surface ectoderm at day E10.5 and secondary fiber cell differentiation begins once the lens vesicle has been occluded with elongated primary fiber cells, at approximately day E12.5. Therefore, it is unclear from the western blot analyses at what point after ablation of *Dlg-1* the changes in Fgfr signaling first occurred.

**Figure 4:**
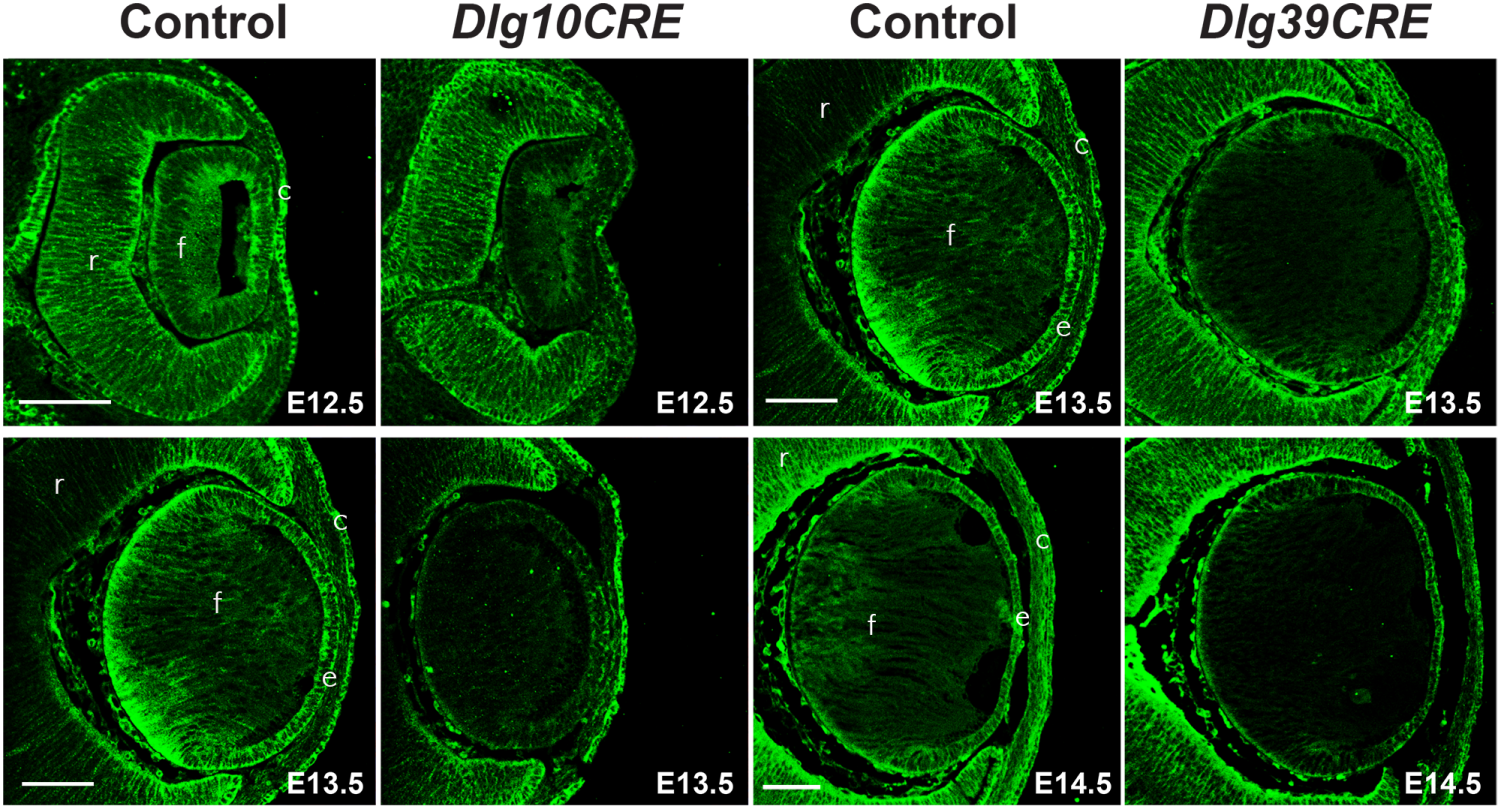
Loss of Dlg-1 protein following cre-mediated excision of *Dlg-1* sequences. Paraffin embedded sections of eyes from control, *Dlg10CRE* and *Dlg39CRE* day E12.5, E13.5, and E14.5 embryos were subjected to immunoflourescent staining for Dlg-1 (green). For *Dlg10CRE* lenses, the intensity of staining throughout the lens was greatly reduced at E12.5 and staining was undetectable at E13.5. For *Dlg39CRE* lenses, the intensity of staining in the fiber cell compartment was greatly reduced at E13.5 and staining in the fiber cell compartment was undetectable at E14.5. c, cornea; e, lens epithelium; f, lens fiber cells; r, retina. Bar = 50 µm.

To determine when loss of Dlg-1 protein occurred in *Dlg10CRE* and *Dlg39CRE* lenses, immunofluorescence experiments were carried out on paraffin embedded sections of E12.5 and E13.5 *Dlg10CRE* and E13.5 and E14.5 *Dlg39CRE* embryos. In keeping previous reports [27], [32], immunoreactivity for Dlg-1 in *Dlg10CRE* lenses was reduced at E12.5 and absent at E13.5 (Fig. 4). Immunoreactivity for Dlg-1 in the fiber cell compartment of *Dlg39CRE* lenses was reduced at E13.5 and absent at E14.5 (Fig. 4).

**Figure 5:**
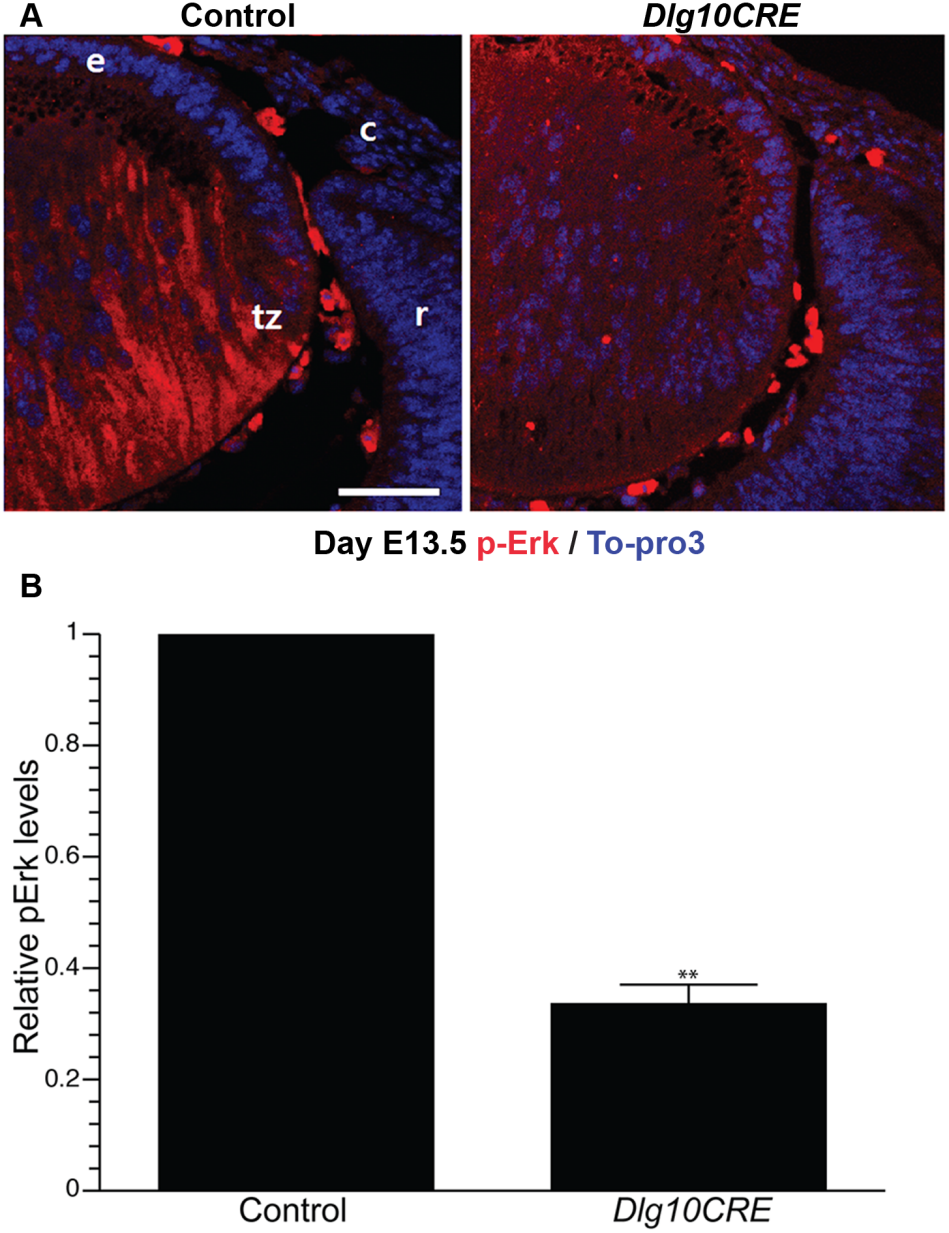
Levels of the Fgfr signaling intermediate, pErk, are reduced in embryonic *Dlg10CRE* lenses. (A) Paraffin embedded sections of eyes from E13.5 control and *Dlg10CRE* embryos were subjected to immunofluorescence analysis using an anti-pErk antibody (red) and the nuclei counterstained with To-Pro3 (blue). Representative images of the transition zone are shown. c, cornea; e, lens epithelium; r, retina; tz, transition zone. Bar = 50 µm. (B) Quantification of pErk levels. Shown are the relative levels of pErk in the transition zone of *DLG10CRE* and control lenses (control levels set at 1.0) Quantification of signal intensities in the transition zone was carried out using ImageJ, as described in Materials and Methods, and the data subjected to statistical analysis using the two-sided One Sample t-test. At least 3 different sections from at least 3 different lenses were evaluated. The relative levels of pErk in the transition zone of the *Dlg10CRE* lens were reduced as compared to levels in the corresponding region of the control lenses. Error bars = standard deviations. * = FDR<0.01.

To determine if Fgfr signaling is compromised at the time Dlg-1 protein is lost from the *Dlg10CRE* lens, paraffin embedded sections from day E13.5 control and *Dlg10CRE* embryos were immunostained with anti-pERK antibodies. The intensities of staining in transition zone of control and *Dlg10CRE* lenses were measured by ImageJ (see Materials and Methods). In control lenses, pErk staining was observed throughout the fiber cell and was notably more concentrated toward the posterior region (Fig. 5). In contrast, the intensity of pErk staining was reduced in *Dlg10CRE* lenses by 64% (Fig. 5). These data show that Fgfr signaling is diminished at the time when Dlg-1 protein is lost from the lens and at a time in lens development much closer to the time when secondary fiber differentiation begins, suggesting that impaired Fgfr signaling does contribute to the observed defects in fiber cell differentiation. Furthermore, as reduced pErk was observed in the transition zone, a region of the lens that exhibits PCP, the data suggest that Dlg-1 may regulate fiber cell differentiation through its role in PCP.

### Effect of Loss of *Dlg-1* on Fgfr2 Levels during Embryogenesis

**Figure 6:**
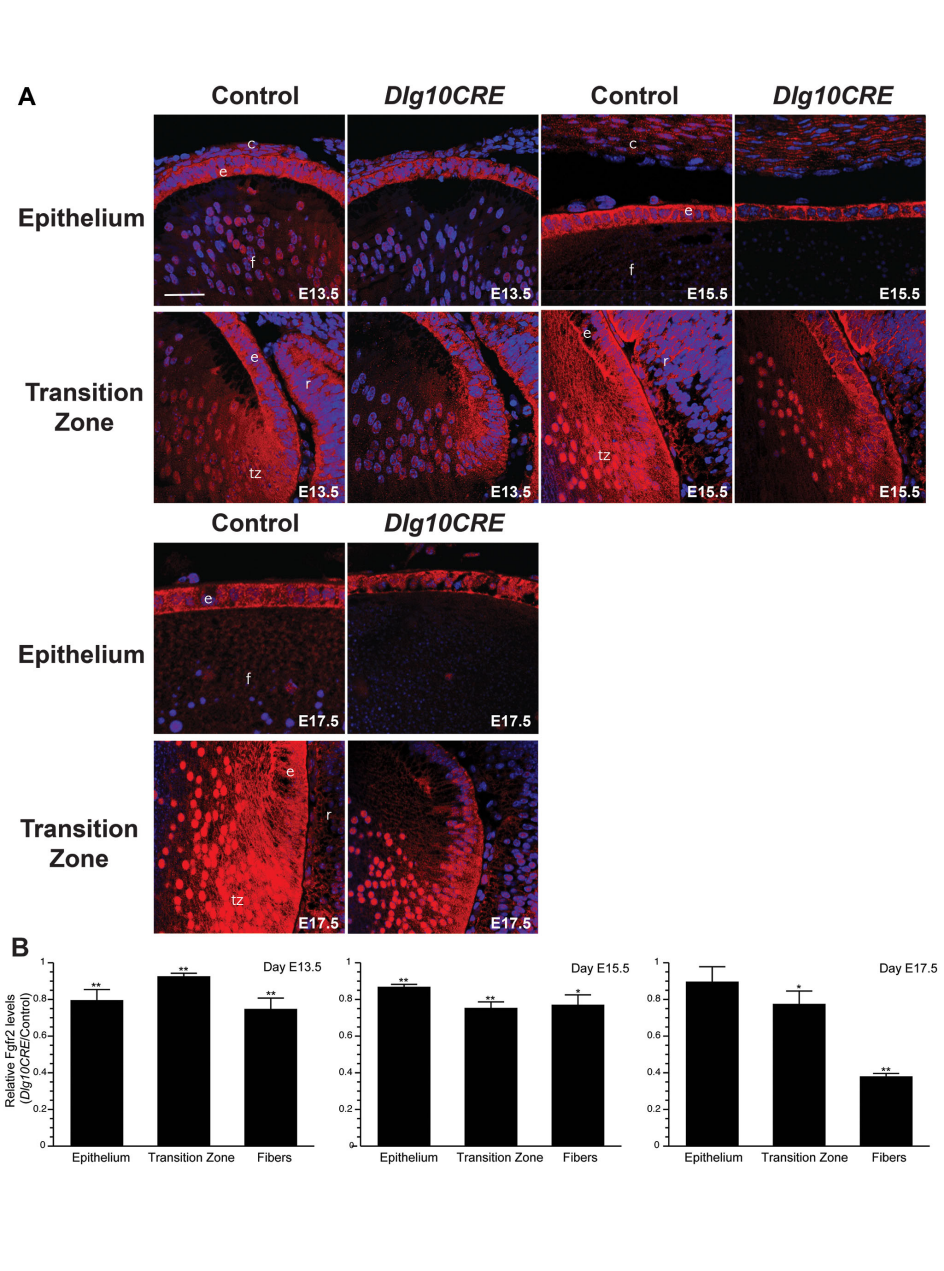
Levels of Fgfr2 are reduced in embryonic *Dlg10CRE* lenses. (A) Paraffin sections of eyes from day E13.5, E15.5 and E17.5 control and *Dlg10CRE* embryos were subjected to immunofluorescence analysis using an anti-FGFR2 antibody (red) and the nuclei counterstained with To-Pro3 (blue). Representative images of the epithelium and transition zone/fiber cell compartment from control and *Dlg10CRE* eyes are shown for each time point. c, cornea; e, lens epithelium; f, lens fibers; r, retina; tz, transition zone. Bar = 50 µm. (B) Quantification of Fgfr2 levels. Shown are the relative levels of FGFR2 in regions of the *Dlg10CRE* lens as compared to levels in the corresponding regions of the control lenses (control levels set at 1.0). Quantification of signal intensities in epithelium, transition zone and fibers was carried out using ImageJ, as described in Materials and Methods, and the data subjected to statistical analysis using the two-sided One Sample t-test. At least 3 different sections from at least 3 different lenses were evaluated for each time point. The Fgfr2 levels were reduced in transition zone and fiber cells of *Dlg10CRE* lenses compared to controls at all time points and in the epithelium of the *Dlg10CRE* lenses compared to controls at E13.5 and E15.5. Error bars = standard deviations. * = FDR<0.05, ** = FDR<0.01.

Given that levels of pErk were reduced in the lenses of E13.5 *Dlg10CRE* embryos, we asked if levels of Fgfr2 were similarly reduced in *Dlg10CRE* lenses at earlier time points. To do so, immunofluorescence experiments were carried out on paraffin embedded sections from control and *Dlg10CRE* E13.5, E15.5, and E17.5 embryos using an anti-FGFR2 antibody. Representative images are shown in Fig. 6A. The intensities of staining in epithelium, transition zone and fiber cells in control and *Dlg10CRE* lenses were measured by ImageJ (see Materials and Methods). In control lenses, staining for Fgfr2 was seen in the epithelium, the transition zone, and the fiber cells (Fig. 6B). Also, Fgfr2 was detected in fiber cell nuclei. The intensity of staining for Fgfr2 in *Dlg10CRE* lenses was significantly reduced at E13.5 in all three areas of the lens (Fig. 6B). Levels of Fgfr2 were similarly reduced at E15.5 and were reduced in the transition zone and fibers at E17.5. The reduced intensity of staining for Fgfr2 in the transition zone and fiber cells appeared to be in both the cell membranes and the nuclei of the *Dlg10CRE* lenses. These results show that Fgfr2 levels begin to decrease at least by a time soon after the beginning of secondary fiber cell differentiation, suggesting that *Dlg-1* deficiency affects Fgfr signaling at the level of the receptors and this contributes to the observed defects in fiber cell differentiation.

### Effect of Loss of *Dlg-1* on Fgfr2 Levels in *Dlg39CRE* Embryonic Lenses

**Figure 7:**
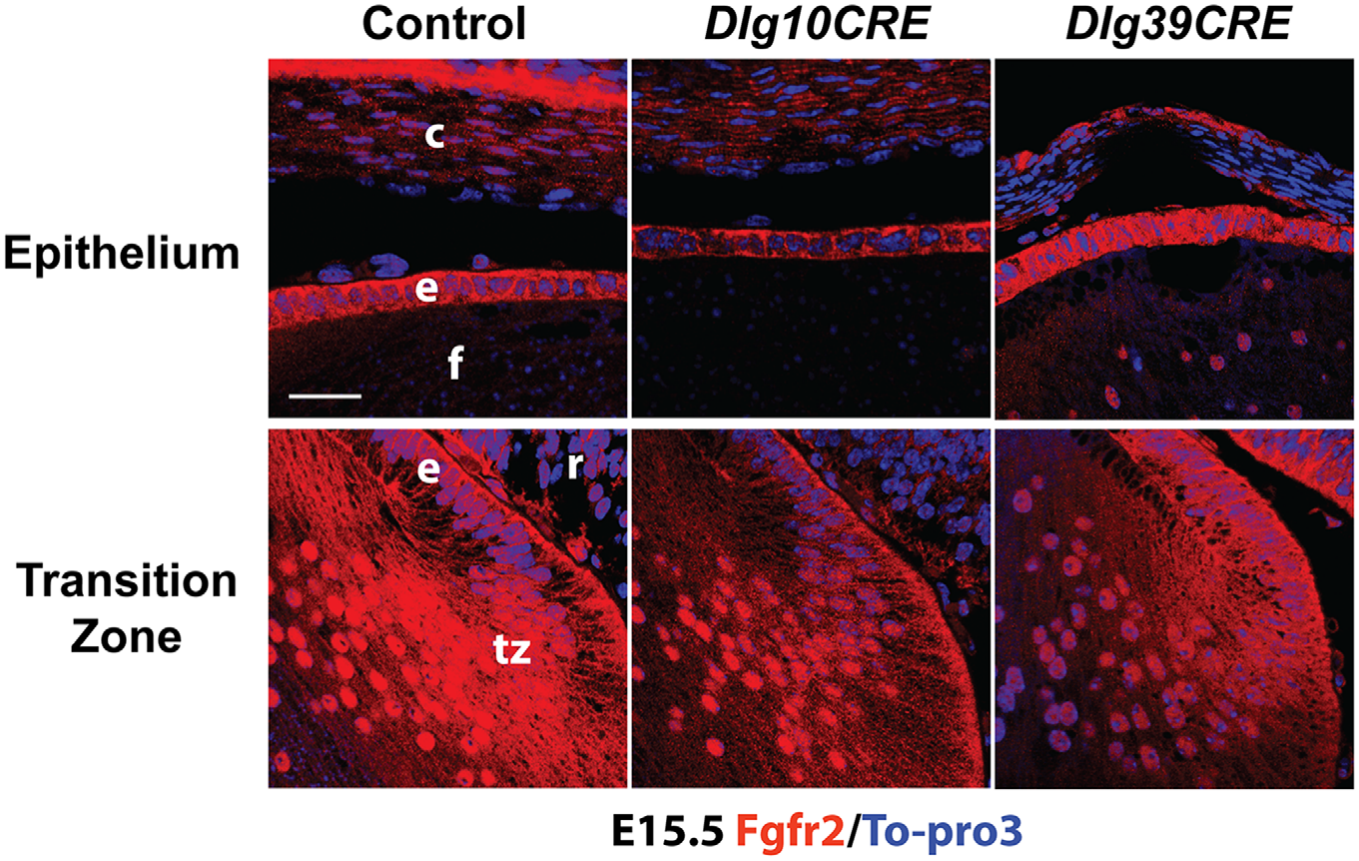
Fgfr2 levels are reduced in the transition zone and fiber cells of *Dlg39CRE* embryos. Paraffin embedded sections from day E15.5 control, *Dlg10CRE* and *Dlg39CRE* embryos were subjected to immunofluorescence analysis using an anti-FGFR2 antibody (red) and the nuclei counterstained with To-Pro3 (blue). Representative images of the epithelium and transition zone of each genotype are shown. The intensity of staining in the epithelium from the *Dlg39CRE* embryo is similar to that in control whereas intensity in the epithelium of *Dlg10CRE* embryos is less than in the control. The intensity of Fgfr2 staining was reduced in the transition zone of both *Dlg10CRE* and *Dlg39CRE* lenses as compared to controls, indicating that the effect is fiber cell autonomous. At least 3 different sections from at least 3 different lenses were evaluated. c, cornea; e, lens epithelium; f, lens fiber cells; r, retina; tz, transition zone. Bar = 50 µm.

In the *Dlg10CRE* lenses, reduced levels of Fgfr2 were found in the epithelium as well as in the fiber cells (Fig. 6). To determine if the effect of *Dlg-1* deficiency on Fgfr2 levels in the fiber cells was independent of the effect in the epithelium, sections from day E15.5 control, *Dlg10CRE* and *Dlg39CRE* embryos were subjected to immunofluorescence experiments using an anti-Fgfr2 specific antibody. As shown in Fig. 7, the intensity of immunofluorescent staining for Fgfr2 in the transition zone of *Dlg39CRE* lenses was reduced as compared to that in the controls whereas the intensity of staining in the epithelium was similar. Therefore, effect of loss of *Dlg-1* on Fgfr2 levels in the fiber cells is a fiber cell autonomous, suggesting that Dlg-1 plays a role in regulating Fgfr2 levels in the fiber cells independent of its role in the epithelium.

### Localization of Dlg-1 and Fgfr2 in Lens Fiber Cells

**Figure 8:**
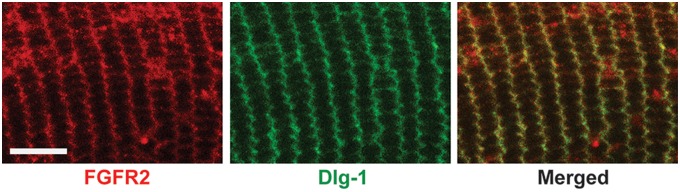
Dlg-1 and Fgfr2 co-localize on the short sides of the fiber cells in the outer cortex. Cryogenic sections from control P30 lenses were subjected to immunofluorescent staining using anti-Fgfr2 anti-Dlg-1 antibodies. Staining for Fgfr2 (red) was predominantly localized on the short sides of the fiber cells as was staining for Dlg-1 (green). Overlap (yellow) can be seen in the merged image. Bar = 50 µm.

PDZ domain containing proteins such as Dlg-1 are thought to function as scaffolding molecules capable of assembling large signaling complexes at the cell membrane [33]. Therefore, it is possible that Dlg-1 modulates Fgfr2 signaling because the two are associated in a large macromolecular complex. To begin to address this possibility, double immunofluorescence experiments were carried out using anti-Dlg-1 and anti-Fgfr2 antibodies on transversely oriented sections from the transition zone region of P30 control mice. Immunostaining for Fgfr2 was predominantly localized to the short sides of the fiber cells in the outer cortex (Fig. 8A), as was immunostaining for Dlg-1 (Fig. 8B). In merged images, overlap in staining was observed (Fig. 8C), suggesting that Dlg-1 and Fgfr2 colocalize on the short sides of the fiber cells.

## Discussion

The development and maintenance of specialized organs is dependent on the ability of cells to coordinate their response to growth factors in the environment with cell structure and the overall architecture of the tissues. In this study, we examined the relationship between Fgfr signaling, which is known to be required for lens fiber cell differentiation [16], and the regulation of cell structure and tissue architecture by *Dlg-1*, a gene involved in both apical-basal polarity and planar cell polarity [24], which has also been shown to be required for fiber cell differentiation [22]. We found that loss of *Dlg-1* led to reduced levels of Fgfr signaling intermediates and downstream targets. Loss of *Dlg-1* also led to changes in the relative abundance of Fgfrs. Finally, loss of *Dlg-1* led to reduced levels of Fgfr2 and pErk, an Fgfr signaling intermediate, in a region of the lens that exhibits PCP. Thus, *Dlg-1* is the first PCP gene to be shown to modulate Fgfr signaling and fiber cell differentiation. That loss of *Dlg-1* impairs Fgfr signaling suggests that the mechanism through which *Dlg-1* regulates fiber cell differentiation is, at least in part, through regulation of Fgfr signaling.

### *Dlg-1* is a Modulator of Fgfr Levels and Fgfr Signaling in Fiber Cell Differentiation

In this study, we showed that *Dlg-1* deficient lenses have reduced levels of numerous activated intermediates of the Fgfr signaling pathway as well as changes in the relative levels of Fgfrs 1, 2 and, to a lesser extent, Fgfr3 compared to control lenses. The changes in Fgfr intermediates may be responsible for some of the observed defects in the *Dlg10CRE* lenses. For example, the reduced levels of pErk may be related to the observed fiber cell elongation defects as this is known to be dependent on pErk [22], [25]. Diminished levels of pAkt may be responsible for the apoptosis observed in the *Dlg10CRE* lenses [22], as it is known that pAkt is necessary for cell survival [34]. We also found reduced levels of pFrs2α, which is directly activated by the Fgfrs [29], [30], and reduced levels of Fgfr2 that was similar to the reduction in levels of pFrs2α (40% vs. 30%), suggesting that the impairment of Fgfr signaling occurs at the level of the receptor. Levels of Fgfr2 and pErk were reduced in *Dlg10CRE* lenses at least by day E13.5, indicating that the effect of loss of *Dlg-1* on Fgfr signaling occurred early in the course of secondary fiber cell differentiation. Interestingly, we found increased levels of Fgfr1, the significance of which is not known at this time. Additionally, the mechanism driving the shift in the relative levels of Fgfrs is not known. Further studies will be required to address these questions. However, in certain breast cancer cell lines, silencing Fgfr2 with siRNA results in increased Fgfr1 levels [35], providing a precedent for our observations.

We found that the subcellular distribution of Fgfr2 differed between epithelium and fiber cells. In the epithelium, the staining was only membrane associated. However, in the fiber cells, staining was associated with both the membrane and the nucleus. In the *Dlg10CRE* fiber cells, levels of Fgfr2 appeared to be reduced in both membrane and nuclear compartments, suggesting multiple functions of Fgfr2 are affected by loss of *Dlg-1*. Shifts in the subcellular distribution of Fgfrs have been observed previously. For instance, in certain cancers, shift in localization of Fgfrs 1 and 2 has been correlated with the stage of the cancer [36], [37]. Perhaps more relevant to lens fiber differentiation, nuclear staining for Fgfr2 has been observed in the terminal end buds of the mammary gland [38] and also in Sertoli cell precursors [39]. The role of nuclear Fgfr2 is unknown at the present time. However, given the circumstances under which nuclear Fgfr2 has been found, it is possible that nuclear Fgfr2 might be important for regulating gene expression tied to entering or maintaining the differentiated state.

### Reduced Fgfr Signaling in *Dlg-1* Deficient Lenses is not Sufficient to Account for Differentiation Defects

Previously, we showed that *Dlg-1* deficiency leads to inhibition of fiber cell differentiation, which was characterized by numerous changes in gene expression and defects in fiber cell structure [22]. Herein, we have determined that loss of *Dlg-1* leads to reduced Fgfr signaling and changes in the relative abundance of the Fgfrs. As Fgfr signaling is known to be required for fiber cell differentiation [1], [2], [16], it is likely that the reduced level of Fgfr signaling in the *Dlg-1* deficient lenses contributes to the observed defects in fiber cell differentiation in these mutant lenses. Fgfr2 has been shown to be required for maintaining normal rates of proliferation in the epithelium, cell cycle withdrawal and cell survival if deletion occurs at the lens placode stage [12]. This phenotype bears some similarity to the *Dlg10CRE* phenotype as *Dlg10CRE* lenses display small changes in rates of proliferation in the epithelium (unpublished data) and apoptosis in both the epithelium and fiber cell compartment of postnatal mice [22], suggesting that these aspects of the *Dlg10CRE* phenotype could be due at least in part to the reduced level of Fgfr2. However, the timing of gene disruption differs between these two models as disruption of *Fgfr2* with *LeCre* occurs at the lens placode stage whereas disruption of *Dlg-1* using *MLR10CRE* occurs only after lens vesicle has formed. It is known that ablating one or two Fgfrs in the lens with *MLR10CRE* does not result in cell cycle or fiber cell differentiation defects [16], suggesting that in the lens the Fgfrs function redundantly or can compensate for each other. Ablating Fgfrs 1, 2, and 3 with *MLR10CRE* results in a near complete failure of fiber cell differentiation [16], a pleiotropic phenotype that differs from that of the *Dlg10CRE* fiber cells. Taken together these data suggest that it is likely that loss of *Dlg-1* disrupts signaling pathways in addition to Fgfr signaling that contribute to the observed phenotype. A number of other growth factor signaling pathways have been reported to affect fiber cell differentiation including BMP [40], [41], TGFβ superfamily [42], [43], Notch [44], [45] and Eph/Ephrin [46]. Additionally, signaling pathways via cell adhesion proteins such as N-cadherin [47] and integrins [48], [49], [50], [51] are known to play roles in lens development. Impairment of any of these signaling pathways could cooperate with defects in Fgfr signaling to elicit the defects in fiber cell differentiation in the *Dlg-1* mutant lenses. Future studies are needed to determine the contribution of altered receptor levels to the *Dlg-1* deficient lens phenotype and identify additional signaling pathways that are modulated by *Dlg-1*.

### Dlg-1 Modulates Fgfr Signaling and Fiber Cell Differentiation through PCP

Recent studies in rat lens explants have shown that Fgf-induced differentiation is dependent on Wnt signaling [19]. This appears to be due to Wnt/PCP signaling for several reasons. First, studies using Wnt/β-catenin reporter mice have shown an absence of activity in the differentiating fibers [19], [52]. Second, blocking Wnt signaling *in vivo* in transgenic mice by overexpressing Sfrp2 inhibits fiber differentiation and results in a phenotype similar to the lens phenotype of mouse mutants in core PCP genes, *Vangl2* and *Celsr1* [17], [18]. Third, in the outer cortical fiber cells, the core PCP proteins are asymmetrically localized and that these cells have a cilium that is oriented toward the anterior pole of the lens, showing that these cells exhibit PCP [17], [18]. The lenses of the *Vangl2* and *Celsr1* mutant mice, *Vangl2^Lp/Lp^* and *Crsh*, respectively, and the Sfrp2 transgenic mice are characterized by a flattened and elongated shape, defects in fiber cell curvature, and defects in suture formation [17], [18]. These same defects are observed in *Dlg-1* mutant lenses [22]. Given that *Dlg-1* plays a role in PCP [24] and the phenotype of the *Dlg-1* mutant lens resembles that of the *Vangl2^Lp/Lp^* and *Crsh* mutant lenses, it is likely that the mechanism through which *Dlg-1* affects Fgfr signaling and fiber differentiation is through its role in PCP. Thus, *Dlg-1* is the first PCP gene that has been shown to modulate Fgfr signaling and lens fiber cell differentiation. A connection between Dlg-1 and Fgf signaling has been suggested previously. During intestinal tube formation in *C. elegans*, DLG-1 interacts with VANG-1 and is phosphorylated by the FGF-like receptor EGL-15 [53]. In the mouse, *Dlg-1* and *Cask* cooperate to regulate kidney development through a mechanism that involves regulation of Fgf8 expression [54]. Together, these and our findings support the concept that there is interaction between Dlg-1 and Fgfr pathways and this interaction has an impact on developmental processes.

Interestingly, we observed that Fgfr2 and Dlg-1 overlap on the short sides of the cortical fiber cells, suggesting that Dlg-1 and Fgfr2 may interact in a complex. This interaction is not likely to be direct as there is no known PDZ binding motif in Fgfr2. Dlg-1 also has been shown to be involved in vesicle trafficking [55], which could play a role in Dlg-1’s modulation of Fgfr2 and PCP proteins. Cell adhesion and cytoskeletal organization are also compromised in the *Dlg10CRE* lenses [22], which could impair PCP and, consequently, Fgfr signaling and fiber cell differentiation. Of note, we have observed that the levels of the cell adhesion protein, N-cadherin, (Lee and Griep, unpublished data) and α-catenin [22] are reduced in *Dlg10CRE* lenses. Future studies will be needed to determine the detailed mechanism through which PCP and Dlg-1 modulate Fgfr signaling and fiber cell differentiation.

## Supporting Information

Figure S1 **Documentation of the specificity of the anti-Fgfr2 antibody.** Paraffin embedded sections of day E17.5 control and *Fgfr2^f/f^;LeCre* embryos were immunostained with anti-Fgfr2 antibodies (red) and counterstained with To-pro3 (blue). Representative images are shown. Immunoreactivity for Fgfr2 in the lens of the *Fgfr2^f/f^;LeCre* embryo was nearly absent while immunoreactivity in the retina was similar between control and *Fgfr2^f/f^;LeCre* embryo. The signal intensity for Fgfr2 staining in the *Fgfr2^f/f^;LeCre* lens, quantified as described in Materials and Methods, was less than 10% of that in the control lens. c, cornea; e, lens epithelium; f, lens fibers; r, retina. Bar = 50 µm.
(TIF)

Figure S2 **Levels of Fgfr2 are reduced in** ***Dlg10CRE*** **lenses.** RIPA lysates of lenses from control and *Dlg10CRE* P2 mice were subjected to western blot analysis for Fgfr2 and the blots reprobed for Gapdh as a loading control. Shown is a representative full western blot of lysates from control and *Dlg10CRE* lenses for Fgfr2. Two independently prepared pools of lysates from control and two independently prepared pools of lysates from *Dlg10CRE* lenses are included on this blot. Lanes: (1) control extract #1, (2) *Dlg10CRE* extract #1, (3) control extract #2, (4) *Dlg10CRE* extract #2, (5) molecular mass markers with sizes (in kD) indicated.
(TIF)
