# Supplementary figures and images for: Loss of Dlg-1 in the Mouse Lens Impairs Fibroblast Growth Factor Receptor Signaling
Source: PLoS One. 2014 May 13;9(5):e97470. doi: 10.1371/journal.pone.0097470 (PMC4019587; doi:10.1371/journal.pone.0097470)

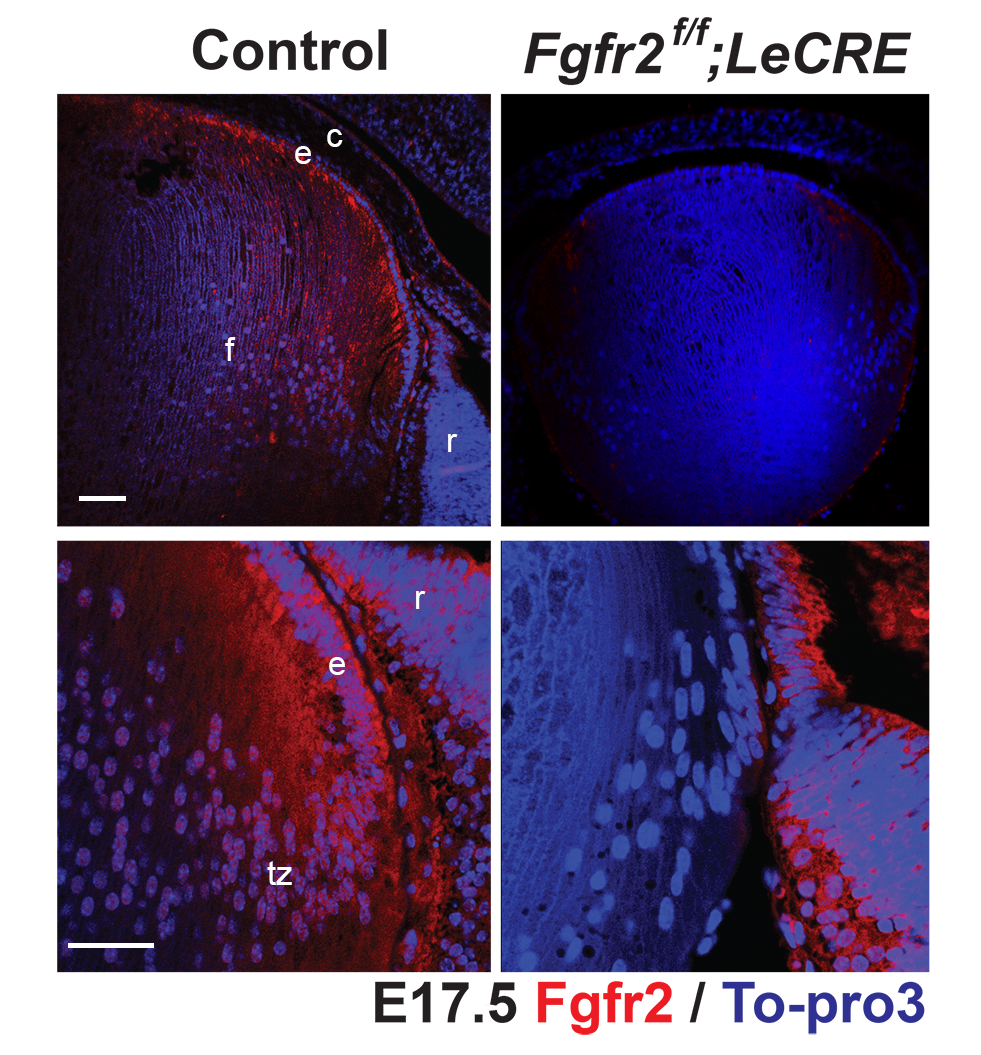

Supplement: Figure S1 — Documentation of the specificity of the anti-Fgfr2 antibody. Paraffin embedded sections of day E17.5 control and Fgfr2f/f;LeCre embryos were immunostained with anti-Fgfr2 antibodies (red) and counterstained with To-pro3 (blue). Representative images are shown. Immunoreactivity for Fgfr2 in the lens of the Fgfr2f/f;LeCre embryo was nearly absent while immunoreactivity in the retina was similar between control and Fgfr2f/f;LeCre embryo. The signal intensity for Fgfr2 staining in the Fgfr2f/f;LeCre lens, quantified as described in Materials and Methods, was less than 10% of that in the control lens. c, cornea; e, lens epithelium; f, lens fibers; r, retina. Bar = 50 µm. (TIF) [file pone.0097470.s001.tif]

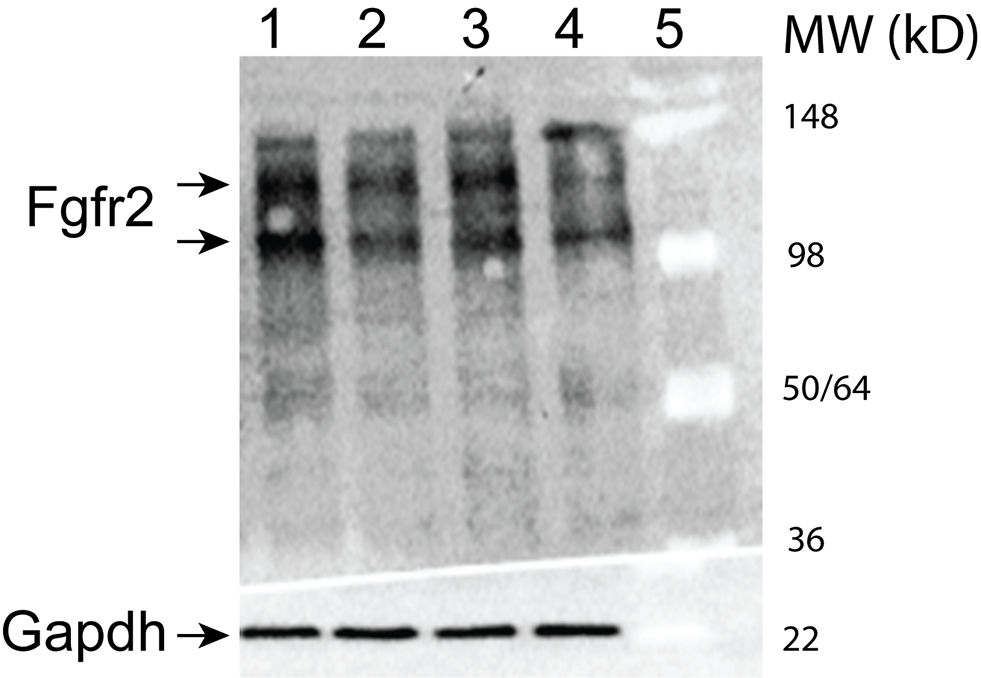

Supplement: Figure S2 — Levels of Fgfr2 are reduced in Dlg10CRE lenses. RIPA lysates of lenses from control and Dlg10CRE P2 mice were subjected to western blot analysis for Fgfr2 and the blots reprobed for Gapdh as a loading control. Shown is a representative full western blot of lysates from control and Dlg10CRE lenses for Fgfr2. Two independently prepared pools of lysates from control and two independently prepared pools of lysates from Dlg10CRE lenses are included on this blot. Lanes: (1) control extract #1, (2) Dlg10CRE extract #1, (3) control extract #2, (4) Dlg10CRE extract #2, (5) molecular mass markers with sizes (in kD) indicated. (TIF) [file pone.0097470.s002.tif]
